# A Desquamating Skin Rash in a Pediatric Patient

**DOI:** 10.5811/cpcem.2019.1.41162

**Published:** 2019-02-26

**Authors:** John Haggerty, Felipe Grimaldo

**Affiliations:** Naval Medical Center San Diego, Department of Emergency Medicine, San Diego, California

## Abstract

Prompt identification and treatment of true dermatologic emergencies is essential in emergency medicine, especially in vulnerable populations such as pediatric patients. This is a case of a three-year-old female who presented with significant dehydration in the setting of a desquamating skin rash diagnosed in our emergency department as staphylococcal scalded skin syndrome.

## INTRODUCTION

Dermatologic emergencies are rare. A five-year, multicenter review found that 3.3% of all emergency department (ED) visits were for general skin complaints, with only 4% of these requiring inpatient admission.^1^ Some rashes are true emergencies, however, and thus require prompt diagnosis and treatment. Such rashes include necrotizing fasciitis, toxic epidermal necrolysis syndrome (TENS), and staphylococcal scalded skin syndrome (SSSS). SSSS will be discussed here.

## CASE REPORT

A previously healthy and fully vaccinated three-year-old female was brought by her parents to the ED with a rash that had been worsening over a five-day period. The mother of the patient reported a fall from playground equipment with resulting abrasion just prior to the onset of the rash. In appearance, the rash was macular, mildly erythematous, and located over the child’s trunk and face. Desquamation of skin surrounding the abrasion occurred after subsequent removal of an adhesive bandage applied to the area. The child had two healthcare visits before presenting to our ED. A few days after the fall, the patient’s primary care physician diagnosed her with an allergic reaction and treated her with diphenhydramine. Further worsening of the rash prompted the parents of the patient to seek care at an outside ED, where she was again diagnosed with an allergic reaction, given diphenhydramine, and also treated with intravenous (IV) fluids. Of note, after this last visit to the ED, further desquamation occurred with removal of adhesives used to secure a peripheral IV on her arm.

**Image 1:**
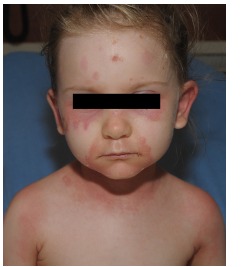
Periorbital and perioral distribution of the edematous, erythematous, macular rash.

**Image 2:**
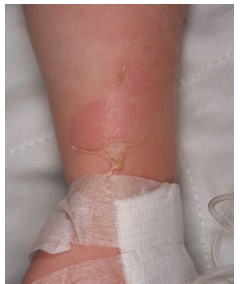
Detail of the left forearm showing desquamation from removal of adhesive tape.

In our department the parents denied fevers but reported decreased per os (PO) intake, with only one episode of urination in the prior 24 hours, along with worsening fatigue. The child also reported some dysuria and odynophagia. She denied respiratory symptoms, vomiting or diarrhea. On physical examination, the child was mildly tachycardic with no other vital sign abnormalities. She appeared fatigued but was interactive during the examination. She had areas of slightly edematous erythema around her periorbital areas, cheeks, neck, upper back, and inguinal area with areas of surrounding desquamation (Images 1 and 2). The original abrasion was surrounded by more pronounced edema and erythema consistent with a small, localized cellulitis. There was no evidence of mucosal involvement on examination of the pharynx and vaginal introitus. The child received fluid resuscitation with a 20 milligram- per-kilogram bolus of IV normal saline, blood cultures were taken, and IV clindamycin initiated. Although there was no mucosal involvement on our examination, there was higher concern for Stevens-Johnson or TENS, since the child reported dysuria and odynophagia. The dermatology service was consulted and recommended admission to the pediatric intensive care unit (PICU) and the addition of ceftaroline to her IV clindamycin. The child was admitted to the PICU, where she received continued IV antibiotics, fluid resuscitation and wound care for the desquamating lesions. By hospital day three, she had no further wound desquamation and had improved urine output. She was discharged after a five-day hospital stay with a seven-day course of PO cephalexin.

## DISCUSSION

SSSS occurs when exfoliative toxins produced by some species of *Staphylococcus aureus* bind and destroy proteins at the granulosum layer of the epidermis, resulting in the characteristic bullae of SSSS and a positive Nikolsky’s sign. In SSSS, this separation of layers occurs within the epidermis as opposed to TENS, where the separation occurs at the dermal-epidermal layer. The more superficial split in the epidermis is one of the key factors in SSSS being a less serious condition than TENS. In bullous impetigo the toxins act locally, whereas in SSSS the toxins spread hematogenously, resulting in desquamation at sites distant to the infectious site. The toxins are excreted via the kidneys, with almost complete clearance in normal renal function, resulting in few cases of SSSS in healthy adults. Children, especially neonates, with developing kidney function, and adults with kidney disease are thus unable to clear the toxins, resulting in higher incidence in these populations.^2^

**CPC-EM Capsule**

What do we already know about this clinical entity?

*Staph scalded skin syndrome (SSSS) is a well- understood, but relatively rare disease which that is often overshadowed by other emergent rashes in clinical teaching*.

What makes this presentation of disease reportable?

*SSSS is a potentially life-threatening rash in vulnerable populations. We describe the classic appearance and distribution of this skin disease*.

What is the major learning point?

*SSSS is endemic to pediatric patients and patients with renal compromise. Prompt diagnosis and treatment can prevent significant morbidity and mortality*.

How might this improve emergency medicine practice?

*Given that hindsight is 20/20, it wasn’t until our patient’s third clinical interaction that SSSS was properly identified. It is essential that emergency physicians be astute diagnosticians*.

The gold standard for diagnosis in SSSS is histology, but clinically the disease may be identified by history and physical. An important diagnostic step is differentiating between SSSS and the more dangerous TENS, which are similar in appearance but differ significantly in mortality. A key clinical differentiation lies in examination of the mucosal membranes. Significant mucosal involvement of the desquamating lesions is a hallmark of TENS, with sparing of the mucosa in SSSS. Of note, SSSS may induce dehydration and therefore dry, cracked lips, but will not induce mucosal desquamation itself. Another key clinical difference between SSSS and TENS is that the more superficial split in the epidermis seen in SSSS leads to a much thinner, more superficial desquamation of the skin, which is much less likely to lead to the fluid loss and risk of secondary infection that is seen in TENS. However, this difference in the thickness of desquamation may be difficult to differentiate clinically for those not familiar with both conditions. Skin biopsy with frozen section analysis can provide definitive differentiation within minutes to hours for those cases where there is any doubt.

Treatment consists of either PO or parenteral IV antibiotics and supportive care. A patient with a small affected area, minimal desquamation, and good PO intake may be treated on an outpatient basis with a seven-day course of dicloxacillin or a cephalosporin.^3^ Patients with larger affected areas of desquamation will need fluid resuscitation and IV antibiotics with likely coverage for methicillin resistant *S. aureus* in the ED. Typical agents might include parenteral vancomycin or ceftaroline IV, but may depend on local resistance patterns. Antibiotic coverage may later be narrowed during hospitalization depending on culture results or clinical course. Clindamycin may be considered as an adjunctive agent due to its unique function as an inhibitor of bacterial toxin production in addition to bacteriostatic action but patterns of resistance have been observed when used alone. 4 Lastly, burn care of desquamated areas is crucial to prevent secondary infections and for patient comfort.

## CONCLUSION

In summary, this case demonstrates a relatively rare desquamating pediatric rash – staphylococcal scalded skin syndrome. Rapid identification and treatment of this disease is essential to avoid secondary infections, sepsis and renal failure.

## References

[1] Baibergenova A, Shear NH (2011). Skin conditions that bring patients to emergency departments. Arch Dermatol.

[2] Patel GK, Finlay AY (2003). Staphylococcal scalded skin syndrome: diagnosis and management. Am J Clin Dermatol.

[3] Habif TP, Habif TP (2015). Bacterial Infections. Clinical Dermatology, A Color Guide to Diagnosis and Therapy.

[4] Braunstein I, Wanat KA, Abuabara K (2014). Antibiotic sensitivity and resistance patterns in pediatric staphylococcal scalded skin syndrome. Pediatr Dermatol.

